# Metabolically and immunologically beneficial impact of extra virgin olive and flaxseed oils on composition of gut microbiota in mice

**DOI:** 10.1007/s00394-019-02088-0

**Published:** 2019-09-10

**Authors:** Jasmine Millman, Shiki Okamoto, Aoki Kimura, Tsugumi Uema, Moeko Higa, Masato Yonamine, Toyotaka Namba, Emi Ogata, Satoru Yamazaki, Michio Shimabukuro, Masato Tsutsui, Masayuki Matsushita, Shinya Ikematsu, Hiroaki Masuzaki

**Affiliations:** 1 Division of Endocrinology, Diabetes and Metabolism, Hematology, Rheumatology, (Second Department of Internal Medicine), Graduate School of Medicine, University of the Ryukyus, Okinawa, Japan; 2 Department of Bioresources Engineering, National Institute of Technology, Okinawa College, Okinawa, Japan; 3 Department of Diabetes, Endocrinology and Metabolism, School of Medicine, Fukushima Medical University, Fukushima, Japan; 4 Department of Pharmacology, Graduate School of Medicine, University of the Ryukyus, Okinawa, Japan; 5 Department of Molecular and Cellular Physiology, Graduate School of Medicine, University of the Ryukyus, Okinawa, Japan

**Keywords:** Gut microbiota, Flaxseed oil, Extra virgin olive oil, Antimicrobial peptide, Regulatory T cells

## Abstract

**Purpose:**

Extra virgin olive oil (EVOO) and flaxseed oil (FO) contain a variety of constituents beneficial for chronic inflammation and cardio-metabolic derangement. However, little is known about the impact of EVOO and FO on dysbiosis of gut microbiota, intestinal immunity, and barrier. We, therefore, aimed to assess the impact of EVOO and FO on gut microbiota, mucosal immunity, barrier integrity, and metabolic health in mice.

**Methods:**

C57BL/6 J mice were exposed to a low-fat (LF), lard (HF), high fat-extra virgin olive oil (HF-EVOO), or high fat-flaxseed oil (HF-FO) diet for 10 weeks. Gut microbiota assessment was undertaken using 16S rRNA sequencing. Levels of mRNA for genes involved in intestinal inflammation and barrier maintenance in the intestine and bacterial infiltration in the liver were measured by qPCR.

**Results:**

HF-EVOO or HF-FO mice showed greater diversity in gut microbiota as well as a lower abundance of the Firmicutes phylum in comparison with HF mice (*P *< 0.05). The qPCR analyses revealed that mRNA level of FoxP3, a transcription factor, and IL-10, an inducer of regulatory T cells, was significantly elevated in the intestines of mice-fed HF-EVOO in comparison with mice-fed HF (*P* < 0.05). The mRNA level of the antimicrobial peptide, RegӀӀӀγ, was markedly elevated in the intestines of HF-EVOO and HF-FO compared with HF group (*P *< 0.05).

**Conclusions:**

Our data suggest that the consumption of EVOO or FO can beneficially impact gut microbiota, enhance gut immunity, and assist in the preservation of metabolic health in mice.

**Electronic supplementary material:**

The online version of this article (10.1007/s00394-019-02088-0) contains supplementary material, which is available to authorized users.

## Introduction

Metabolic health can be described as the body’s ability to successfully balance fuel oxidation and fuel availability in an endocrine-influenced environment [1]. In today’s current obesogenic climate, however, attaining this metabolic balance is becoming ever more difficult to achieve, and as a consequence, the burden of lifestyle-related metabolic diseases is rapidly increasing worldwide. Low-grade, sustained systemic inflammation, considered to lie at the heart of metabolic dysfunction, is strongly linked to the consumption of obesogenic diets high in pro-inflammatory-saturated fats [2, 3]. Chronic intake of diets high in saturated fats not only leads to inevitable weight gain, but can also compromise intestinal barrier function, causing metabolic endotoxemia, inducing systemic inflammation partly through toll-like receptor (TLR) signaling [4].

A number of mechanisms employed by the mucosal immune system aim to assist in the protection of gut barrier and preservation of host defense. Antimicrobial peptides (AMPs) are one such example. Primarily produced by Paneth cells, however, also by enterocytes in the intestine, they create a physical barrier, separating gut microbiota from intestinal cells, employing a variety of bacteriostatic and bactericidal mechanisms [5]. For example, RegӀӀӀγ, a representative AMP of the Lectin family, uses non-enzymatic attack to destroy pathogenic bacteria through the TLR–myeloid differentiation primary response 88 (MyD88)-signaling pathway via recognition of microbiota-associated molecular patterns (MAMPs), eventually promoting its transcription and subsequent production [5]. The adaptive immune system also plays a fundamental role in modulating the local gut environment and composition of microbiota, with key players such as intestinal regulatory T (Treg) cells, aiding mucosal tolerance, and enforcing commensalism [6, 7].

It has been expeditiously recognized that the gut microbiota is crucial in both mediating host inflammation and influencing host metabolic health, with diet as a critical factor in determining the diversity and function of the microbial community [8]. A diet high in saturated fatty acids (SFAs) and low in dietary fibers is considered to be one of the main factors driving dysbiosis of gut microbiota, decreasing microbial diversity and beneficial species of bacteria as well as increasing the abundance of certain pathogenic species of bacteria, overall negatively impacting metabolic health in both humans and rodents [9].

To date, however, little is known about the potential impact of specific plant-derived dietary fats and oils on composition of gut microbiota and host metabolic health. Extra Virgin Olive oil (EVOO) and Flaxseed oil (FO), two major oils marketed as ‘functional foods,’ contain a wide variety of compounds purported to have anti-inflammatory properties and beneficial effects on markers of metabolic health including blood glucose and lipids, body weight, and inflammation [10, 11]. EVOO, a staple in the mediterranean diet, contains appreciable amounts of monounsaturated fatty acids (MUFAs) and also a variety of phenolic compounds which possess antioxidant, anti-inflammatory, and anti-bacterial activity that are transformed into bioactive metabolites by resident gut microbiota [12, 13]. In comparison, FO is an abundant source of α-linolenic acid (ALA), an essential *ω*3 polyunsaturated fatty acid (PUFA), but contains only a small quantity of phenolic compounds [11, 12].

In this context, the present study aims to characterize the potential impact of EVOO and FO on mouse gut microbiota, with a particular focus on gut barrier integrity, mucosal immunity, and metabolic health—specifically in relation to fuel homeostasis, inflammation, and gut permeability. Our hypothesis is that the ingestion of either EVOO or FO will exert superior effects on mouse gut microbiota composition and diversity, intestinal immune function, and metabolic health in comparison with mice-fed HF or even LF diets.

## Materials and methods

### Animals and diets

6-week-old male C57BL/6 J mice were obtained from Charles River Laboratories Japan, Inc. (Kanagawa, Japan) and were housed (5 mice per cage) at 24 °C under a 12 h/12 h light/dark cycle. All animal care and experimental procedures were approved by the Animal Experiment Ethics Committee of the University of the Ryukyus (No. 5352, 5718 and 5943). Mice were allowed to acclimatize for 2 weeks prior to the start of the dietary intervention and were fed ad libitum with water and a low-fat purified diet. After 2 weeks of acclimatization, mice (*n* = 5 per group) were randomly allocated to receive one of four experimental diets (Research Diets, New Brunswick, NJ, USA). Experimental diets were as follows; low-fat, purified (LF) (10% energy from fat), saturated fat (HF) (45% energy from fat, 35% from lard), extra virgin olive oil (HF-EVOO) (45% energy from fat, 35% from EVOO), and flaxseed oil (HF-FO) (45% energy from fat, 35% from FO). A low-fat, purified diet was used in replace of a grain-based standard chow to control the effect of dietary fiber on gut microbiota with an increased proportion of corn starch and maltodextrin used as opposed to an increased proportion fat used in other diets. In all diets, sucrose and protein were matched. Macronutrient distribution of the experimental diets is shown in Table 1. Composition regarding major fatty acids composition in the experimental diets is shown in Table 2.

**Table 1:** Macronutrient distribution in the experimental diets

	LF		HF		HF-EVOO		HF-FO	
%	G	Kcal	g	kcal	g	kcal	g	kcal
Protein	19	20	24	20	24	20	24	20
Carbohydrate	67	70	41	35	41	35	41	35
Fat	4	10	24	45	24	45	24	45
Total		100		100		100		100

	LF		HF		HF-EVOO		HF-FO	
Ingredient	g	kcal	g	kcal	g	kcal	g	kcal
Corn starch	452.2	1809	72.8	291	72.8	291	72.8	291
Maltodextrin	75	300	100	400	100	400	100	400
Sucrose	172.8	691	172.8	691	172.8	691	172.8	691
Cellulose	50	0	50	0	50	0	50	0
Soybean oil	25	225	25	225	24.8	223.4	24.8	223.4
Flaxseed oil	0	0	0	0	0	0	157.8	1420
Extra virgin	0	0	0	0	157.8	1420	0	0
olive oil								
Lard	20.33	183	177.6	1598	20.3	183	20.3	183
Total	1055.4	4060	858.2	4057	858.5	4061	858.5	4061

LF diet contained 3.8 kcal/g of energy and HF, HF-EVOO and HF-FO diets contained 4.7 kcal/g of energy, respectively

**Table 2:** Major fatty acid composition of Lard, EVOO, and FO used in the experimental diets

Major fatty acid	LF	HF	HF-EVOO	HF-FO
16:0 Palmitic acid	14.4	18.2	12.1	7.2
18:0 Stearic acid	6.9	9.8	4.0	4.2
18:1 Oleic acid (MUFA)	27.4	31.7	63.0	19.1
18:3 Linoleic	39.5	27.8	15.8	21.1
18:3 α-Linolenic acid (n-3 PUFA)	4.7	2.1	1.6	46.1
SFA	23.7	31.7	17.3	12.3
MUFA	30.0	35.6	64.5	19.9
PUFA	46.3	32.7	18.0	67.8
Trans-fatty acid	0	0	0.2	0.1

% of total fat

Mice had free access to each of the diets for a period of 10 weeks. Body weight and food intake were measured and recorded weekly. To assess diet-related changes in metabolic markers, on the morning following last day of the dietary treatment phase, mice were sacrificed by decapitation. Liver, intestinal tissue samples, and caecum contents were collected immediately and flash frozen in liquid nitrogen. Whole blood was collected into heparinized tubes and plasma was obtained after centrifugation at 4 °C, at 880×*g* for 15 min. All samples were stored at − 80 °C until further processing.

### DNA extraction and 16S rRNA sequencing

Microbial DNA was isolated from caecum contents using QIAamp Fast DNA Stool Mini Kit (QIAGEN, Tokyo, Japan) as per manufacturer’s instructions. PCR amplification was performed using 16S rRNA universal primers targeting the V3–V4 region; 341F (TCGTCGGCAGCGTCAGATGTGGTATAAGAGACAGCCTACGGGNGGCWGCAG) and 806R (GTCTCGTGGGCTCGGAGATGTGTATAAGAGACAGGGACTACHVGGGTWTCTAAT). Sequencing fragments were detected and analyzed using MiSeq Illumina platform (Illumina, San Diego, CA). Raw sequences were filtered using the QIIME software package (Version 1.9.1). Amplicon reads were clustered into operational taxonomic units (OTUs) at 97% identity threshold and aligned against Greengenes reference database (Version 13.5). A total of 193 representative sequences (most abundant OTUs) were aligned using Greengenes reference database and taxonomically assigned the Ribosomal Database project Classifier. Microbial alpha diversity was measured by phylogenetic diversity (PD) whole tree, Chao1, observed OTUs and Shannon indices and rarefaction plots generated using QIIME. Microbial beta diversity was displayed as Principal Coordinates Analysis (PCoA) plots based on unweighted UniFrac distances using Emperor, [14] displaying the relative relatedness of species among groups and the percentage variance among groups explained by the principal coordinate axes. Phylogenetic Investigation of Communities by Reconstruction of Unobserved States (PICRUSt) was used to predict differences in metagenome functions using OTUs generated from 16S rRNA-sequencing data [15]. Normalized OTUs for Kyoto Encyclopedia of Genes and Genomes (KEGG) orthologs were then used to predict functions based on gene counts for each sample.

### Analyses of quantitative RT-PCR

Total RNA was extracted from liver, ileum, and proximal colon samples using Trizol reagent (Thermo Fisher, Carlsbad, CA) and quantified using nanodrop (Thermo Fisher, Scientific). The cDNAs were synthesized using an iScript cDNA Synthesis Kit (Bio-Rad, Hercules, CA) according to the manufacturers’ instructions. Quantitative real-time PCR was performed using Applied Biosystems StepOneplus Real-Time PCR systems (Applied Biosystems) and SYBR Green (Takara Bio, Shiga, Japan), following the manufacturers’ instructions. All samples were analyzed in duplicate and mRNA levels normalized to those of 18S rRNA. The following quantitative PCR conditions were as follows: 95 °C for 10 min, followed by 40 cycles of 95 °C for 15 s and 60 °C for 60 s. The primer sets used for quantitative real-time PCR analysis are shown in the Supplementary Materials section (Supplementary Table S1).

### Analyses of plasma short-chain fatty acids (SCFAs)

SCFAs (acetate, propionate, butyrate, and valerate) in plasma were measured using LC–MS/MS. Standard SCFA solutions (Merck, MO, USA) were used for generating calibration curves. Acetic acid-*d*_*4*_ (FUJIFILM Wako Pure Chemical Corporation, Osaka, Japan) was used as an internal standard. Samples were first subject to deproteinization and SCFAs derived using 2-nitrophenylhydrazine. SCFA derivatives were then extracted by methyl *tert*-butyl ether and injected into the LC–MS/MS system using ACQUITY UPLC system (Waters, Milford, MA, USA) equipped with an analytical column (AQUITY HSS T3 2.1 × 150 mm, 1.8 μm, Waters). Electrospray ionization was carried out with the API4000 (AB Sciex, Foster City, CA, USA) operating in negative ionization and multiple reaction-monitoring mode. The multiple reaction-monitoring transitions labeled for acetic acid, propionic acid, butyric acid, valeric acid, and acetic acid-*d*_*4*_ were *m/z* 194-164, *m/z* 208-178, *m/z* 222-192, *m/z* 236-206, and *m/z* 197-93, respectively.

### Measurements of metabolic parameters

At the end of the 10-week dietary intervention, blood glucose levels, using blood taken from tail vein, were determined using One Touch glucose analyzer (Lifescan, Japan). Both plasma triglyceride (TG) and total cholesterol (TC) levels, with samples obtained by decapitation, were measured using the Cholesterol E kit and Triglyceride E kits (Wako, Japan), respectively, according to manufacturer’s instructions.

### Statistical analyses

Data are shown as mean ± SEM. One-way analysis of variance (ANOVA) followed by multiple comparison tests (Post Hoc, Tukey’s) were used to analyze differences between groups. Non-parametric Kruskal–Wallis tests were used to compare differences in microbiota compositional data among groups. Associations between plasma metabolic markers and microbiota data were examined using linear regression statistics. Levels of statistical significance were set at *: *P *< 0.05, **: *P *< 0.01, ***: *P *< 0.001, and ****: *P *< 0.0001. Statistical analyses were performed in Graph Pad Prism, version 8.0 (Graph Pad Software, San Diego, CA, USA).

## Results

### EVOO and FO prevent obesity and metabolic dysfunction

**Fig. 1:**
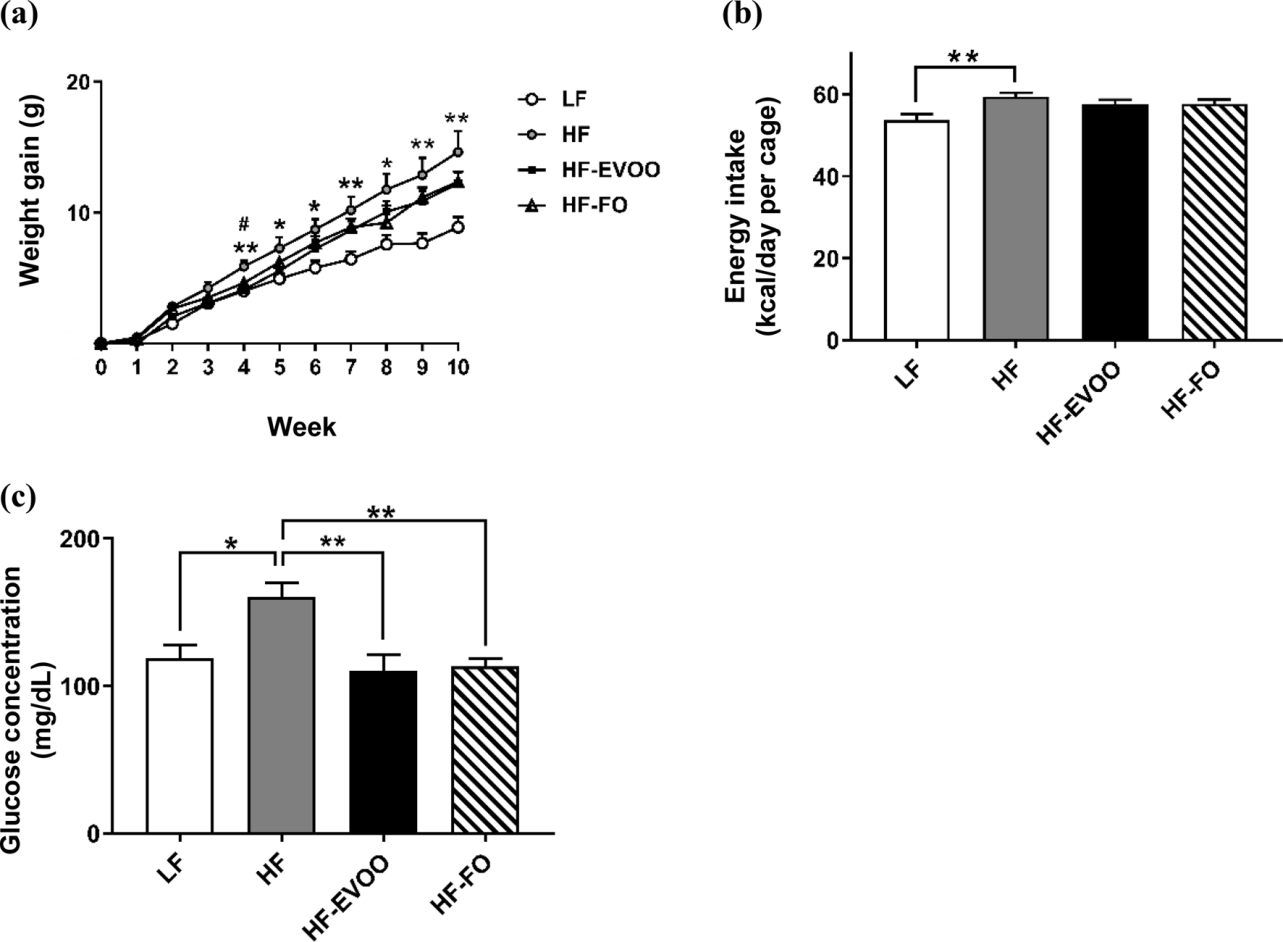
Selected metabolic parameters in mice-fed LF, HF, HF-EVOO, and HF-FO diets. **a** Body weight gain (*Indicates difference between HF and LF; #Indicates difference between HF and HF-EVOO groups), **b** average energy intake and **c** blood glucose levels when fed ad libitum (taken from tail vein). All data were analyzed using one-way ANOVA followed by post hoc Tukey’s multiple comparison test. Data indicate mean ± SEM. *n *= 5 mice per group. *P < 0.05; **P < 0.01

We tested whether consumption of EVOO or FO would beneficially impact weight gain, energy intake, and fuel homeostasis during and at the completion of the 10-week dietary intervention. From 4 weeks, a significant increase in body weight was observed in HF group compared to LF group which continued throughout the 10-week intervention (*P *< 0.05) (Fig. 1a). HF-EVOO- and HF-FO-fed mice also exhibited an increase in body weight in comparison with mice-fed LF throughout the 10-week period. However, there were no significant differences between two groups. A significant increase in energy intake was seen only in HF compared to the LF-fed mice throughout the 10-week intervention (*P* = 0.0081) (Fig. 1b). At the end of the 10-week intervention, a significant increase in blood glucose level was observed in HF group compared to LF (*P *= 0.0214), HF-EVOO (*P *= 0.0054), and HF-FO groups (*P *= 0.0085) (Fig. 1c). In addition, mice-fed HF-EVOO and HF-FO showed a trend to decrease plasma TG and TC in comparison with mice-fed HF. However, differences were not statistically significant (Fig. S2c, d).

### EVOO and FO influence production of microbial-derived metabolites

**Fig. 2:**
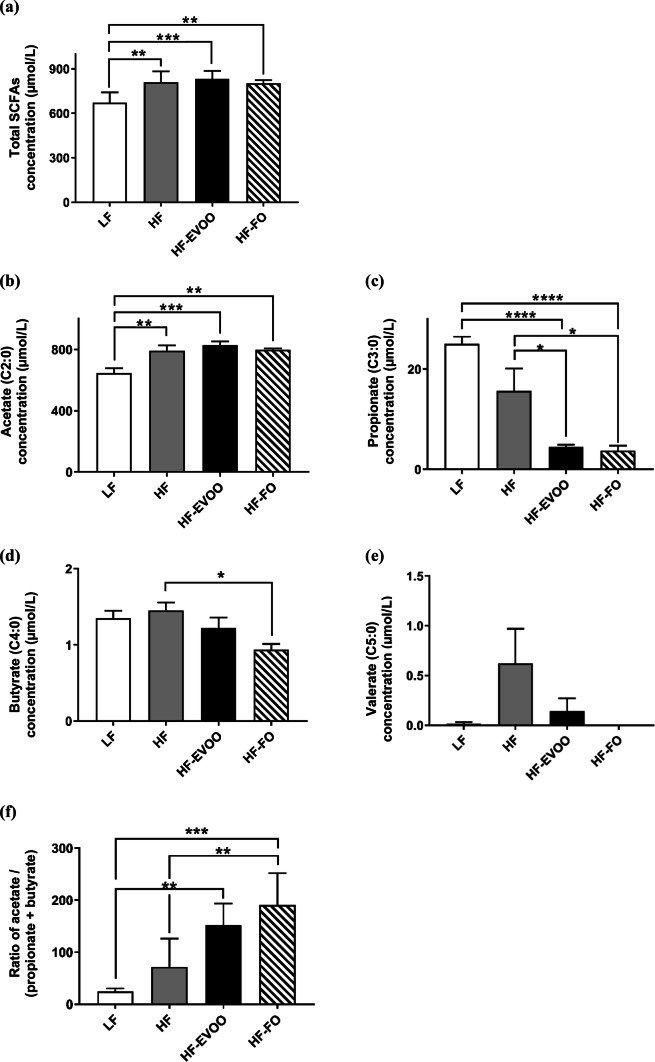
Plasma SCFA profile of mice-fed LF, HF, HF-EVOO, and HF-FO diets. **a** Total concentration of plasma SCFAs, **b–e** individual plasma SCFA concentrations (acetate, propionate, butyrate, and valerate, respectively) and **f** plasma ratio of acetate to butyrate plus propionate. Data indicate mean ± SEM. All data were analyzed using one-way ANOVA followed by post hoc Tukey’s multiple comparison test. *n *= 5 mice per group. *P < 0.05; **P < 0.01, ***P < 0.001; ****P < 0.0001

Total concentration of SCFAs in plasma was significantly increased in HF (*P *= 0.0082), HF-EVOO (*P* = 0.0022), and HF-FO (*P *= 0.0128)-fed mice in comparison with LF-fed mice (Fig. 2a). Higher levels of acetate, the most abundant SCFA, were observed in mice-fed HF (*P *= 0.0064), HF-EVOO (*P *= 0.0009) and HF-FO (*P *= 0.0049) vs. mice-fed LF (Fig. 2b). Propionate concentration in plasma was significantly decreased in HF-EVOO (*P* = 0.0220, *P* < 0.0001) and HF-FO (*P *= 0.0144, *P *< 0.0001) mice in comparison with mice-fed HF and LF, respectively (Fig. 2c). Plasma levels of butyrate were significantly elevated in HF mice vs. HF-FO (*P *= 0.0168) mice; however, levels of this SCFA were scarce (Fig. 2d). Plasma concentration of the minor SCFA, valerate, was elevated in mice-fed HF as well as mice-fed HF-EVOO, with a very small amount detected in mice-fed LF and no detectable levels observed in mice-fed HF-FO diets (Fig. 2e).

Ratio of acetate to butyrate plus propionate, a proposed marker of gut microbiota balance contributing to anti-obesity effects [16, 17], was significantly elevated in HF-FO (*P *= 0.0002, *P *= 0.0039) mice as compared with LF and HF mice, respectively (Fig. 2f). This ratio was also significantly elevated in HF-EVOO (*P *= 0.0022) mice compared to LF mice.

### EVOO and FO impact the composition of gut microbiota and microbial diversity

**Fig. 3:**
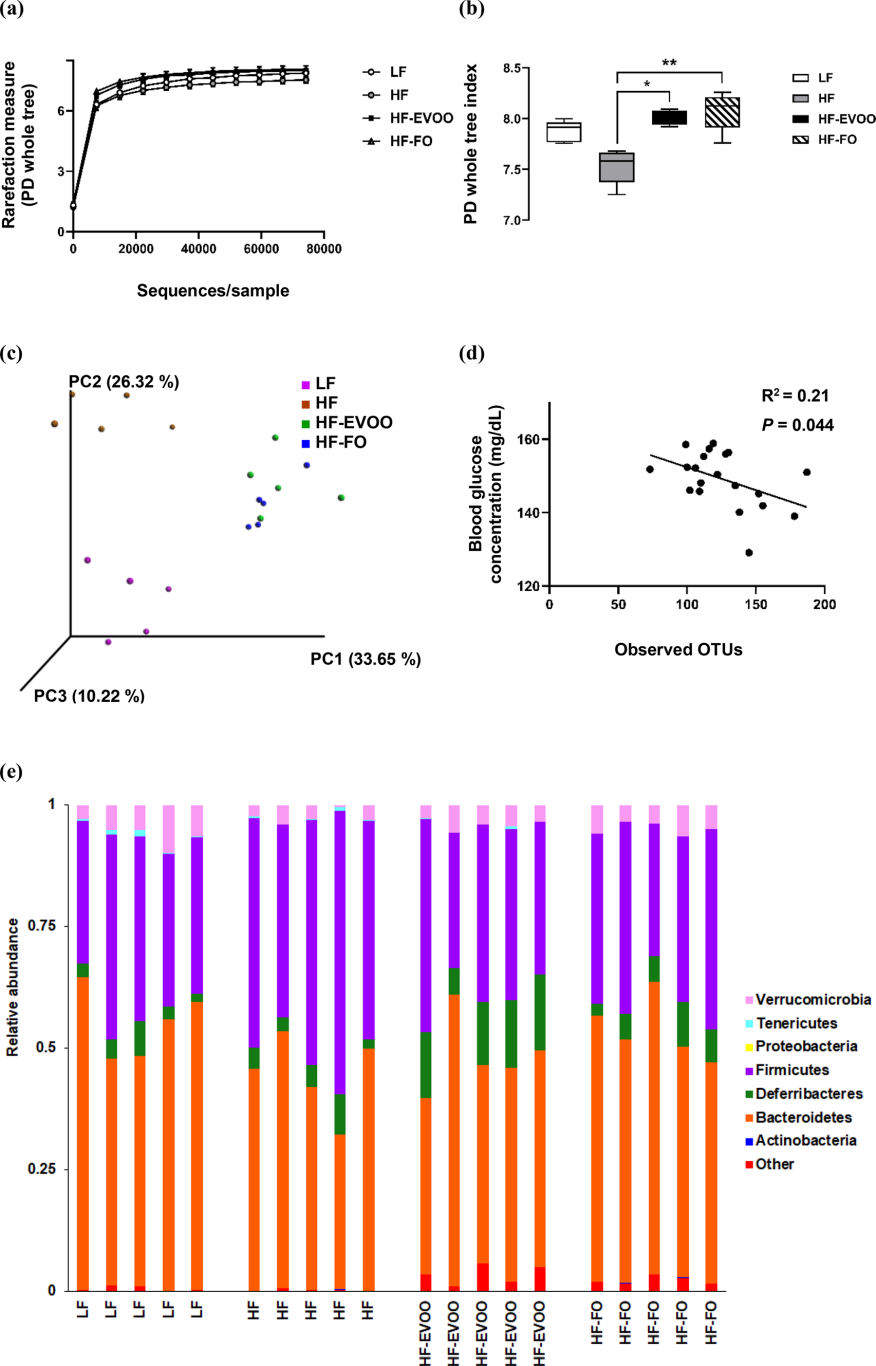

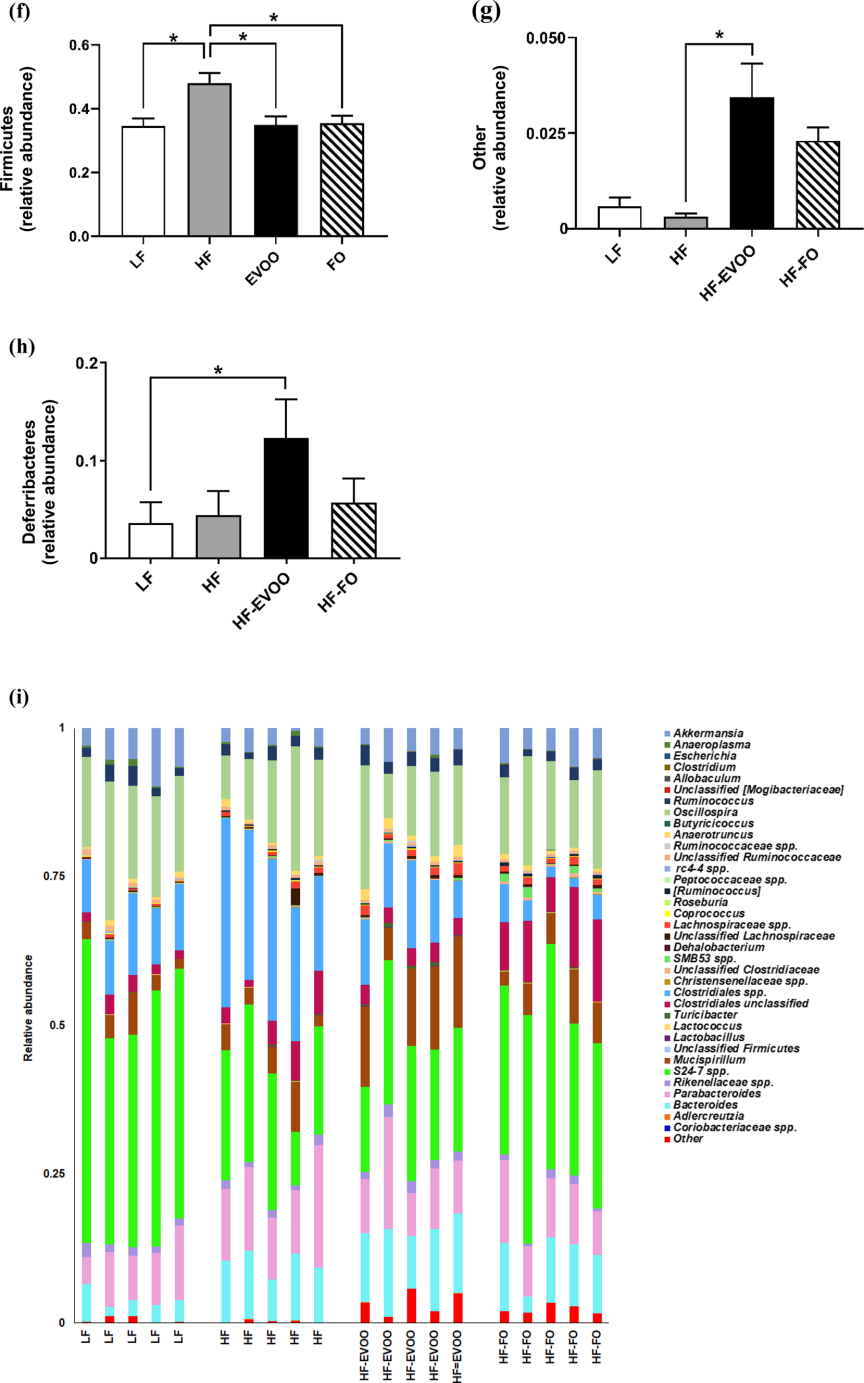

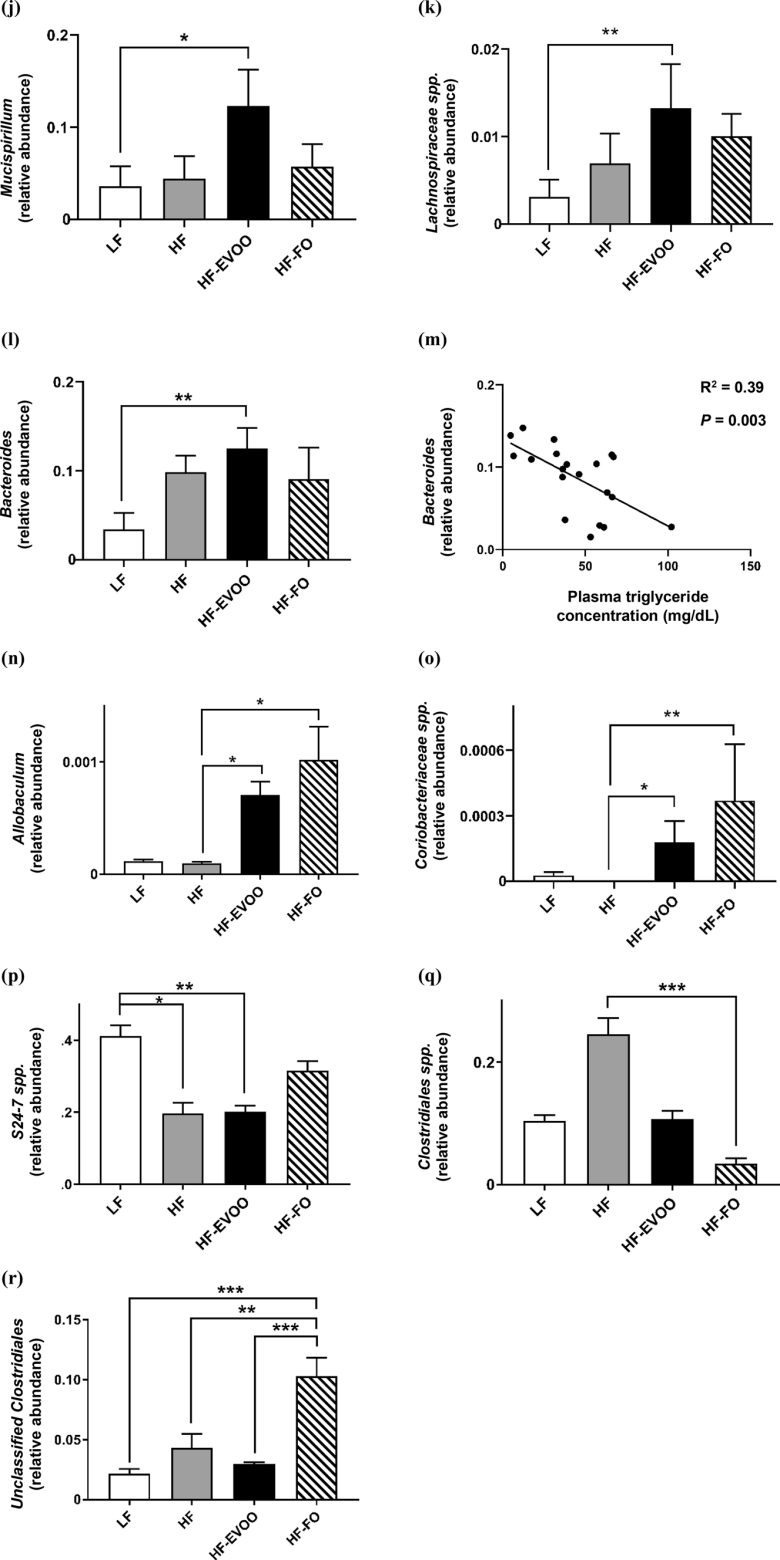
Diversity and composition of gut microbiota in mice-fed LF, HF, HF-EVOO, and HF-FO diets. **a** Rarefaction curve for PD, **b** box plot showing alpha diversity measured by PD, **c** principal coordinate analysis of gut microbiota composition based on unweighted UniFrac, **d** correlation of observed OTUs with blood glucose levels, **e** relative abundance of major phyla composition, **f–h** relative abundance of selected phyla highlighting significant differences among groups, **i** relative abundance of major genera and **j–r** relative abundance of selected genera highlighting significant differences among groups, **m** correlation of *Bacteroides* genera with plasma TG concentration. All data were analyzed using non-parametric, Kruskal–Wallis test. Data indicate mean ± SEM. *n *= 5 mice per group *P < 0.05; **P < 0.01, ***P < 0.001

At the end of the 10-week period, microbiota analysis using caecum contents revealed a number of distinct changes in microbial community structure in mice-fed HF-EVOO and HF-FO diets in comparison with mice-fed HF or LF. Alpha diversity (diversity within samples) was markedly affected in mice-fed HF-EVOO and HF-FO, showing a significant increase in PD in comparison with mice-fed HF diet (Fig. 3a, b). Specifically, the rarefaction curve in Fig. 3a showing PD (units) (*y-*axis) increasing (particularly in HF-EVOO and HF-FO groups) as sampling depth increases (x-axis), with the curve plateauing when maximum sampling depth has been reached. The box plot in Fig. 3b showed a significant increase in PD (units) (*y-*axis) between HF-EVOO and HF (*P *= 0.0327), and HF-FO and HF (*P *= 0.0031)-fed mice. Such increments in alpha diversity observed in HF-EVOO and HF-FO groups also showed similar trends when employing other diversity metrics: Chao1, observed OTUs, and Shannon indexes (Figs. S4, S5). In addition, principal coordinates analysis (PCoA) using unweighted UniFrac distances, a distance measure for comparison of microbial communities, with the percentage of variation explained by PC1, PC2, and PC3, revealed distinct clustering of samples based on respective diets. Specifically, HF-EVOO and HF-FO showed closer similarities compared to LF and HF diets, highlighting that changes in microbial communities among groups were dependent largely on the quantity of different oil/fat consumed (Fig. 3c). Interestingly, richness in microbiota (observed OTUs) showed an inverse correlation with blood glucose levels (Fig. 3d).

Taxonomy-based analysis of relative abundance of gut microbiota (Fig. 3e), with each column representing an individual sample from one of the dietary groups, revealed significantly higher relative abundance of Firmicutes (depicted in purple) in HF vs. LF (*P* = 0.0125), HF-EVOO (*P* = 0.0148), and HF-FO groups (*P* = 0.0184) (Fig. 3f). Higher relative abundance of other phyla (depicted in red) and Defferibacteres (depicted in green) was also observed in mice-fed EVOO in comparison with HF and LF groups, respectively. Specifically, a higher abundance of the other phylum, comprising a number of rarer, less abundant yet important bacterial species, was observed in mice-fed HF-EVOO in comparison with mice-fed HF (*P *= 0.0166) (Fig. 3g). In addition, a significant elevation in the average relative abundance of Defferibacteres was observed in mice-fed HF-EVOO compared to mice-fed LF (*P *= 0.0197) (Fig. 3h). Although the abundance of the phylum Verrucomicrobia was relatively high among groups (Fig. 3e, depicted in pink), significant differences were observed between HF and LF groups only (*P* = 0.0245).

A number of significant differences were also observed in mice-fed HF-EVOO and HF-FO in comparison with mice-fed LF or HF diets at the genus level (Fig. 3i, Supplementary Table S2). A select number of bacterial genera were significantly elevated in mice-fed HF-EVOO in comparison with mice-fed LF (Figs. 3j–l). Specifically, significantly higher abundance of bacteria from the *Mucispirillum* genera was observed in mice-fed HF-EVOO vs. mice-fed LF (*P *= 0.0197) (Fig. 3j). This significant increase in *Mucispirillum* was endorsed by findings at the phylum level showing the increased abundance in Defferibacteres phylum observed in mice-fed HF-EVOO vs. mice-fed LF was driven entirely by *Mucispirillum* at the genus level. A higher abundance of the bacteria from the genera *Lachnospiraceae* (*P *= 0.0080) and *Bacteroides* (*P *= 0.0037) was also observed in HF-EVOO-fed mice in comparison with LF-fed mice (Fig. 3k, l). The increased abundance of *Bacteroides* was associated with lower levels of plasma TG (*R*^2^ = 0.39, *P *= 0.003) (Fig. 3m).

An increased abundance of bacteria from the *Allobaculum* and *Coriobacteriaceae* genera was also observed in mice-fed HF-EVOO (*P *= 0.0234, *P *= 0.0107) and HF-FO (*P *= 0.0139, *P *= 0.0018), respectively, in comparison with mice-fed HF (Fig. 3n, o). Moreover, a significantly lower abundance in bacteria from carbohydrate-degrading genera, *S24*-*7 spp*., (Bacteroidetes phylum), was observed in HF-EVOO (*P *= 0.0097) and HF (*P *= 0.0139) groups compared to LF group (Fig. 3p). Lower levels in the abundance of *Clostridiales spp.* (Firmicutes phylum), associated with obesity and increased in mice-fed HF lard diet [18], was observed in FO-fed mice in contrast to mice-fed HF (*P *= 0.0005) (Fig. 3q). In comparison, bacteria from the *Unclassified Clostridiales* genera (Firmicutes phylum) dominated in mice-fed HF-FO compared to mice-fed LF (*P *= 0.0001) (Fig. 3r). A significant difference in this genus was also observed in mice-fed HF-EVOO (*P *= 0.0004) and HF (*P *= 0.0028) vs. HF-FO.

### EVOO increases the expression of genes involved in the accumulation of intestinal Treg

**Fig. 4:**
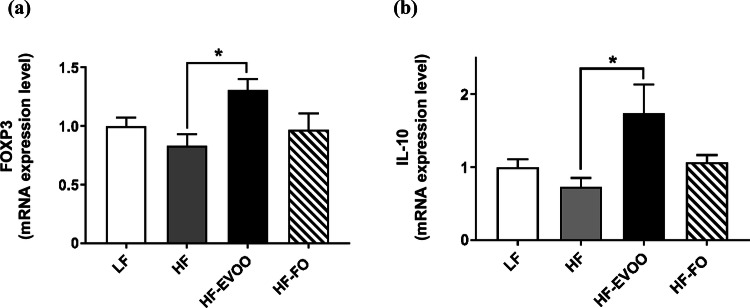
Relative mRNA level in proximal colon of mice-fed LF, HF, HF-EVOO, and HF-FO diets for **a** FOXP3 and **b** IL-10. All data were analyzed using one-way ANOVA followed by post hoc Tukey’s multiple comparison test. Data indicate mean ± SEM. *n *= 5 mice per group. *P < 0.05

To investigate whether Treg expansion may occur in the proximal colon of mice fed either HF-EVOO or HF-FO, we measured Treg-related gene expressions such as Forkhead box P3 (FoxP3) and Interleukin10 (IL-10). The mRNA level for FoxP3, a transcription factor critical for the development of Treg, was significantly elevated in the proximal colon of mice-fed HF-EVOO compared with mice-fed HF (*P* = 0.0125) (Fig. 4a). The level for IL-10, both produced by as well as an inducer of Treg, was also significantly increased in mice-fed HF-EVOO as compared with mice-fed HF (*P *= 0.0136) (Fig. 4b).

### EVOO and FO influence gut barrier and potentially upregulate intestinal antimicrobial defense mechanisms

**Fig. 5:**
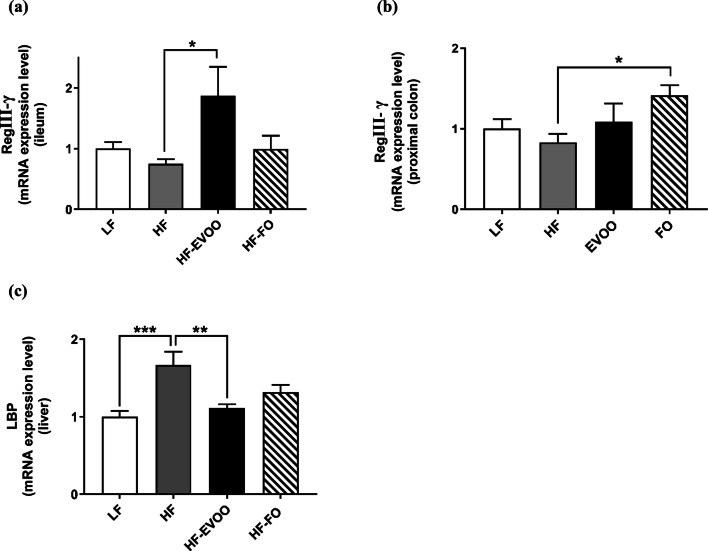
Relative mRNA level of **a** RegӀӀӀγ in ileum, **b** RegӀӀӀγ in proximal colon and **c** lipopolysaccharide-binding protein (LBP) in liver of mice-fed LF, HF, HF-EVOO, or HF-FO diets. All data were analyzed using one-way ANOVA followed by post hoc Tukey’s multiple comparison test. Data indicate mean ± SEM. *n *= 5 mice per group. *P < 0.05; **P < 0.01; ***P < 0.001

RT-PCR was performed to determine mRNA levels of genes involved in AMP production in the ileum and proximal colon as well as bacterial infiltration in the liver. The mRNA expression of RegӀӀӀγ in the ileum was significantly elevated in mice-fed HF-EVOO in comparison with mice-fed HF (*P *= 0.0295) (Fig. 5a). Similarly, the level in the proximal colon was also increased HF-FO mice vs. mice-fed HF (*P* = 0.0475) (Fig. 5b). Hepatic mRNA expression of lipopolysaccharide-binding protein (LBP) was markedly increased in the HF group in comparison with LF (*P *= 0.0005) and HF-EVOO (*P *= 0.0041) groups (Fig. 5c).

## Discussion

The present study clearly demonstrates that mice-fed HF-EVOO and HF-FO exhibited a variety of beneficial effects on markers of health. Specifically, HF-EVOO- and HF-FO-fed mice exemplified a significantly positive impact on blood glucose compared to HF-fed mice. Noticeably, these beneficial effects on glucose homeostasis were observed despite no significant differences in body weight gain or energy intake among groups. In light of this finding, specific components within EVOO and FO, including the *ω*9 and *ω*3 fatty acids from EVOO and FO, respectively, may be partially accountable for such a favorable impact. In accordance with our results, Oliveira et al. recently reported that the substitution of diets with FO and olive oil (OO) for obese mice on a high fat diet resulted in beneficial effects on glucose homeostasis, and found that this effect was partially mediated via *ω*9 and *ω*3 fatty acids from these oils [12]. Briefly, *ω*9 and *ω*3 fatty acids were shown to increase glucagon-like peptide-1 (GLP-1) in the intestine, reverse insulin resistance as well as exert anti-inflammatory activities through G-protein-coupled receptor (GPR) 120 and GPR 40 pathways in liver, skeletal muscle, and adipose tissue [12]. In agreement with this notion, mice-fed HF-EVOO and HF-FO showed a trend to lower levels of plasma TG and TC compared to mice-fed HF.

The present study demonstrates that plasma concentrations of SCFAs differed significantly among groups. Total SCFA concentration as well as the major SCFA, acetate, was significantly lower in mice-fed LF in comparison with mice-fed HF-EVOO, HF-FO, and HF diets. Although the fiber content in all experimental diets was equal, the present study used an LF purified diet as opposed to a grain-based standard chow containing significantly more fiber, in turn leading to the increased production of SCFA, which is commonly observed in animals on the standard chow diets [19]. On the other hand, propionate concentration in plasma was much lower in both HF-EVOO- and HF-FO-fed mice in comparison with LF and HF mice. Considering a recent study demonstrating that higher circulating level of propionate is implicated in exaggerated gluconeogenesis and insulin resistance in both mice and humans [20], our data support the notion that the SCFA profile of mice-fed HF-EVOO and HF-FO is metabolically beneficial. Concentrations of butyrate were significantly lower in HF-FO mice compared to HF mice, although amounts of this SCFA were scarce. The ratio of acetate to butyrate plus propionate in plasma typically increased in lean subjects [16, 17], was significantly lower in mice fed both HF and LF diets in comparison with mice-fed HF-EVOO and HF-FO. These data are partly consistent with a previous report of Nishitsuji et al. demonstrating that not only a trend to increase in the ratio of Firmicutes to Bacteroidetes (Fig. S6), but also the ratio was significantly lower in mice that develop obesity–diabetes syndrome [16]. Despite LF mice exhibiting less body weight gain than HF mice, the lower ratio observed in the LF group is likely the consequence of a low-fat purified diet being used, contributing to lower levels of acetate in particular.

A substantial impact on the composition of gut microbiota was observed in mice-fed EVOO and FO. HF-EVOO and HF-FO-fed mice displayed significantly higher diversity in gut microbiota compared to HF-fed mice as well as β-diversity plots revealing clear differences between HF-EVOO and HF-FO groups vs. LF and HF groups. These findings are in line with the previous reports that a diet high in saturated fats and low in dietary fibers, as consumed by HF-fed mice in the present study, lowers total gut microbial gene count and adversely affects microbial diversity [21, 22]. The present study demonstrates that microbial richness also showed an inverse correlation with blood glucose levels. In mice-fed HF-EVOO and HF-FO, higher levels of microbial diversity and lower blood glucose levels were observed compared to HF mice. In line with this finding, a recent cohort study reported that individuals with low bacterial richness show more insulin resistance and other metabolic abnormalities in comparison with individuals with high bacterial richness [23].

The differing constituents within EVOO and FO may be responsible for their positive effects on gut microbiota diversity, yet likely operate under different mechanisms. For example, the higher diversity in gut microbiota observed in HF-EVOO mice compared to HF mice may also be partially due to the presence of certain phenolic compounds within EVOO (Supplementary Table S3), and their ability to act as prebiotics, stimulating the growth of beneficial bacterial species and impacting microbial diversity [24]. In contrast, the presence of the *ω*3 fatty acids, α-linolenic acid (ALA) in FO, and its ability to be converted into the more potent *ω*3 fatty acid, docosahexaenoic acid (DHA), may play a role in increasing microbiota diversity observed in HF-FO mice vs. HF mice [25]. Although it is well known that majority of dietary fats are indeed digested and absorbed in the small intestine, there is evidence that dietary fats, in particular, MUFAs and PUFAs, can indeed enter the large intestine, potentially exerting effects on gut tissue as well as interacting with resident microbiota [26]. Our data support that this notion in that lipids were extractable from caecum contents of mice and subsequent measurements of TG and TC in stool revealed that diets richer in fats (HF, HF, EVOO, and HF-FO) showed higher levels of these lipids in comparison with mice-fed LF (Fig. S2a, b). Interestingly, a significant elevation in TC in caecum contents was observed in mice-fed HF-EVOO vs. LF. This difference may be partially explained by the presence of polyphenols in EVOO that have been shown to impair cholesterol absorption in the intestine by reducing micellar solubility [27]. In addition, KEGG-functional analyses showed that a number of pathways involved in lipid metabolism that were significantly enriched in HF-EVOO- and HF-FO-fed mice compared to either HF or LF groups, providing further evidence of the presence of fats in the caecum of these mice, and their potential to interact with microbiota (Fig. S3).

In the present study, at the phylum level, a significantly lower relative abundance of the Firmicutes phylum was seen in LF, HF-EVOO, and HF-FO groups in comparison with HF group. An increased abundance of Firmicutes, and lower levels of Bacteroidetes, have been observed in both mice and human subjects with obesity [28]. The other phylum of bacteria as well as Deferribacteres was observed in HF-EVOO-fed mice vs. LF-fed mice. Other phylum of bacteria contains a numerous number of unclassifiable rarer species of bacteria, contributing to gut microbiota diversity. The Deferribacteres phylum contains the genus *Mucispirillum*, which was significantly higher in HF-EVOO mice as compared with LF mice. This bacterium, a core member of the mouse gut microbiome, associates intimately with the mucosal tissue, influencing gene expression and playing a key role in induction of Treg [29, 30]. A recent study by Campbell et al. demonstrated that when the border-dwelling bacteria *Mucispirillum* was depleted in Treg-deficient mice, a subsequent increase in type 2 responses occurred, negatively altering epithelial homeostasis [30, 31]. They also noted that the growth and expansion of *Mucispirillum* were in turn enhanced by Treg by promoting immune tolerance to this bacterium, subsequently shaping the microbiota [30]. In accordance with these previous reports, we also observed a substantial elevation in mRNA level of FoxP3, a transcription factor produced by Treg, in the proximal colon of mice-fed HF-EVOO in comparison with HF-fed mice [32, 33]. In addition, a higher abundance of bacteria from the *Lachnospiraceae* and *Bacteroides* genera, also capable of increasing Treg [7], was observed in mice-fed HF-EVOO in comparison with mice-fed LF. Furthermore, bacteria from the *Bacteroides* genera also showed an inverse association with plasma TG levels, highlighting the possibility that an elevation of this genera observed in HF-EVOO mice may have led to the lower levels of plasma TG observed in this group. This genus also was found to negatively correlate with plasma TG levels in a large, recent cohort study [34]. Moreover, in both HF-EVOO and HF-FO mice, a significant elevation of bacteria from *Allobaculum* and *Coriobacteriaceae*, which are associated with lean phenotype [16], was observed in comparison with HF mice. It should be noted that mice-fed HF-FO showed a significantly higher abundance of bacteria from the *Unclassified clostridiales* genera in comparison with LF, HF, and HF-EVOO. Bacteria belonging to this genus express bile acid-inducible (bai) genes and generate secondary bile acids which exert wide spread effects on host health including antimicrobial effects [35]. Importantly, our KEGG data demonstrated that expression level of bai genes is apparently increased in the microbiota of mice-fed HF-FO as reflected in the augmentation of pathways for both primary and secondary bile acid synthesis, particularly in comparison with HF mice (Fig. S3). Although mechanisms remain to be elucidated, an increase in this genera of bacteria and subsequent enrichment in genes involved in pathways of bile acid production would be a key impact factor of HF-FO consumption on gut microbiota in comparison with other dietary groups.

In the ileum, mice-fed HF-EVOO exhibited a significantly higher level of RegӀӀӀ-γ mRNA in comparison with HF-fed mice, while mice-fed HF-FO showed significantly higher levels of RegӀӀӀ-γ mRNA in the proximal colon compared to mice-fed HF. The RegӀӀӀ-γ protein produced in response to specific Gram-positive, pathogen-causing bacteria, rapidly kills or inactivates bacteria, shaping the bacterial community [5, 36]. Previous studies reported a significant reduction in RegӀӀӀγ expression in both the small intestine and colon under HF and obese conditions [36]. Bacterial–epithelial interactions initiated by lipopolysaccharide and nucleotide-binding oligomerization domain containing molecules (NODs) stimulate MyD88-TLR signaling, thereby activating innate lymphoid cells (ILCs) and Th17 cells and regulating RegӀӀӀγ expression [37–39]. Unequal distribution of Paneth cells throughout the murine intestinal tract as well as distinct defense mechanisms operating in the small vs. large intestine are possible factors contributing to the difference observed RegӀӀӀγ mRNA expression in the ileum of HF-EVOO-fed mice vs. the proximal colon of mice-fed HF-FO [37, 38]. Such an apparent difference between HF-EVOO- and HF-FO-fed mice may be attributable to the distinct constituents within the oil-targeting specific regions of the intestine, modulating not only gut microbiota, but also influencing specific intestinal cell types such as Paneth vs. epithelial cells. Along with RegӀӀӀγ, mRNA expression of MyD88 and IL-22 also showed a trend to increase in mice-fed HF-EVOO and HF-FO (Fig. S8). Notably, IL-22, an interleukin produced by innate ILCs and Th17 cells, modulates epithelial cell function and plays a critical role in licensing epithelial cells for RegӀӀӀγ production [5]. Recently, Fatkhulina et al. uncovered an atheroprotective role of IL-22 via its ability to regulate production of AMPs such as RegӀӀӀγ and prevent the expansion of species of bacteria with pro-atherogenic properties [40]. Specifically, these species of bacteria deliver pro-atherogenic metabolites such as LPS due to a defective intestinal barrier, causing chronic low-grade inflammation and atherosclerosis [40]. In accordance with this notion, we also observed significantly lower levels of LBP mRNA in the liver of mice-fed EVOO or FO in comparison with mice-fed HF diet and a trend for plasma LBP to show higher levels in HF mice, indicating pathophysiologic aggravation of intestinal permeability and subsequent bacterial infiltration in mice-fed HF (Fig. S7). Moreover, an increasing trend in mRNA level of intestinal barrier markers, in particular, ZO-1 and Occludin, was also observed in mice-fed EVOO or FO (Fig S9).

Collectively, our data provide novel evidence that the consumption of EVOO and FO can beneficially impact the composition of gut microbiota, increase microbial diversity as well as influence the production of microbial-derived metabolites contributing to the activation of mucosal immune system, enhancement of markers of gut barrier integrity, and ultimately support metabolic health in mice. Further studies are warranted to test this hypothesis in human clinic-based dietary interventions.

## Electronic supplementary material

Below is the link to the electronic supplementary material.

Supplementary material 1 (PDF 436 kb)
