# Supplementary material for: Metabolically and immunologically beneficial impact of extra virgin olive and flaxseed oils on composition of gut microbiota in mice
Source: Eur J Nutr. 2019 Sep 10;59(6):2411–25. doi: 10.1007/s00394-019-02088-0 (PMC7413911; doi:10.1007/s00394-019-02088-0)
Supplement: Supplementary file 1 — Supplementary material 1 (PDF 436 kb) [file 394_2019_2088_MOESM1_ESM.pdf]

Supplementary materials

Title: Metabolically and immunologically beneficial impact of extra virgin olive and flaxseed oils on composition of gut microbiota in mice

Jasmine Millman[1], Shiki Okamoto[1], Aoki Kimura[2], Tsugumi Uema[1], Moeko Higa[1], Masato Yonamine[1], Toyotaka Namba[1], Emi Ogata[3], Satoru Yamazaki[3], Michio Shimabukuro[3], Masato Tsutsui[4], Masayuki Matsuhita[5], Shinya Ikematsu[2], Hiroaki Masuzaki[1]

[1]Division of Endocrinology, Diabetes and Metabolism, Hematology, Rheumatology (Second Department of Internal Medicine), Graduate School of Medicine, University of the Ryukyus, Okinawa, Japan
[2]Department of Bioresources Engineering, National Institute of Technology, Okinawa College, Okinawa, Japan
[3]Department of Diabetes, Endocrinology and Metabolism, School of Medicine, Fukushima Medical University, Fukushima, Japan
[4]Department of Pharmacology, Graduate School of Medicine, University of the Ryukyus, Okinawa, Japan
[5]Department of Molecular and Cellular Physiology, Graduate School of Medicine, University of the Ryukyus, Okinawa, Japan

Corresponding authors:
Hiroaki Masuzaki
hiroaki@med.u-ryukyu.ac.jp
Tel: +81-98-895-1146, Fax: +81-98-895-1415
ORCID: 0000-0002-2445-1047

Shiki Okamoto
shiki@med.u-ryukyu.ac.jp
Tel: +81-98-895-1146, Fax: +81-98-895-1415
ORCID: 0000-0002-2802-0527

Keywords: Gut microbiota, Flaxseed oil, Extra virgin olive oil, Antimicrobial peptide, Regulatory T-cells

Analysis of phenolic compounds

Qualitative and quantitative determination of phenolic compounds in extra virgin olive oil and flaxseed oils used in the experimental diets were carried out using liquid chromatography (LC) coupled to mass spectrometry (MS) by OP Biofactory, Okinawa, Japan. A total of 18 reference standards were purchased; hydroxytyrosol, tyrosol, vanillic acid, caffeic acid, homovanillic acid, *p*-coumaric acid, benzoic acid, *o*-coumaric acid, cinnamic acid, secoisolariciresinol diglucoside (SDG), oleuropein, luteloin, pinoresinol, kaempferol, apigenin, 1-acetoxypinoresnol and matairesinol. Reserpine was used as the internal standard (IS). Oil samples (750mg) were pre-treated with (10µL) reserpine (IS), acetronile (1mL) was added and samples were subject to sonication and centrifugation. Samples were then extracted two times using methanol (1mL), sonication and centrifugation. Extracts were then pooled and washed twice hexane. The hexane portion was then removed by centrifugal evaporation and the solution resuspended in methanol (100 µL). Extracts (2µL) were then injected for LC/MS analysis and separation was performed on a CORTECS UPLC T3 column (50 × 2.1 mm I.D., 1.6 mm). Elution was carried out at a flow rate of 0.8 mL/min using water/formic acid (A) and acetronile/formic acid (B), at a gradient from 2.5-100% (B) over 13 minutes. Detection was performed at 210 – 500 nm using positive or negative mode electrospray ionization.

Measurement of triglyceride and cholesterol in caecum contents

Lipid extraction from 70-250mg of caecum contents was performed using the Folch method (Folch et al., 1957). Triglyceride (TG) and total cholesterol (TC) concentrations were subsequently measured from lipid extract using Cholesterol E kit and Triglyceride E kits (Wako, Japan).

Supplementary Table S1 Quantitative PCR Primer sequences

**Primers**	**Forward Sequence**	**Reverse Sequence**
18S rRNA	TTCTGGCCAACGGTCTAGACAAC	CCAGTGGTCTTGGTGTGCTGA
FoxP3	CCCATCCCCAGGAGTCTTG	ACCATGACTAGGGGCACTGTA
IL-10	CTGGACAACATACTGCTAACC	GGGCATCACTTCTACCAGGTA
TGF-β	GTCTGGGACCCTGCCCCTAT	TTGCAGGAGCGCACAATCAT
LBP	GTCCTGGGAATCTGTCCTTG	CCGGTAACCTTGCTGTTGTT
ZO-1	CTTCTCTTGCTGGCCCTAAA	TGGCTTCACTTGAGGTTTCTG
OCLN	CACACTTGCTTGGGACAGAG	TAGCCATAGCCTCCATAGCC
CLDN-1	GATGTGGATGGCTGTCATTG	CCTGGCCAAATTCATACCTG
RegIII-γ	TTCCTGTCCTCCATGATCAAA	CATCCACCTCTGTTGGGTTC
MyD88	TGCCGTCCTGTCTACATCTTTG	GTTGCTCAGGCCAGTCATCA

Supplementary Table S2 Relative abundance of genera highlighting significant differences between groups

	**LF**	**HF**	**HF-EVOO**	**HF-FO**	***P* value**
Other	0.005938	0.003145	0.03437	0.02304	0.0166 (HF-EVOO vs. HF)
***Bacteroides* (Bacteroidetes)**	0.03428	0.09847	0.1249	0.09039	0.0037 (HF-EVOO vs LF)
***S24-7 spp.* (Bacteroidetes)**	0.3158	0.2011	0.1968	0.4129	0.0097 (HF-EVOO vs. LF), 0.0139 (HF vs. LF)
***Mucispirillum* (Deferribactres)**	0.03631	0.04421	0.1231	0.05735	0.0197 (HF-EVOO vs. LF)
***Lactococcus* (Firmicutes)**	0.0005945	0.0008650	0.0003536	0.001077	0.0116 (HF-FO vs. HF-EVOO)
***Turicibacter* (Firmicutes)**	0.0003248	0.001256	0.004246	0.001117	0.0007 (HF-EVOO vs. LF)
***Clostridiales unclassified* (Firmicutes)**	0.02170	0.04315	0.02976	0.1029	0.0001 (HF-FO vs. LF), 0.0028 (HF vs. LF), 0.0004 (HF-FO vs. HF-EVOO)
***Clostridiales spp.* (Firmicutes)**	0.1045	0.2450	0.1071	0.03473	0.0005 (HF-FO vs. HF)
***Christensenellaceae spp.* (Firmicutes)**	0.0001094	0.0002953	0.000194	0.0002765	0.0452 (HF-FO vs. LF), 0.0197 (HF vs. LF)
***Unclassified Clostridiaceae* (Firmicutes)**	0.0007045	0.0009122	0.001815	0.004755	0.0025 (HF-FO vs. LF), 0.0234 (HF-FO vs. HF)
***SMB53 spp.* (Firmicutes)**	0.001064	0.001405	0.001746	0.009968	0.0452 (HF-FO vs. LF)
***Lachnospiraceae spp.* (Firmicutes)**	0.003115	0.006964	0.01324	0.01004	0.0080 (HF-EVOO vs. LF)
***Ruminococcus* (Firmicutes)**	0.0007221	0.002259	0.002566	0.00416	0.0021 (HF-FO vs. LF)
***rc4-4 spp.* (Firmicutes)**	0.0006925	0.0001148	0.0008005	0.0006623	0.0277 (HF-FO vs. HF) 0.0452 (HF-EVOO vs. HF)
***Allobaculum* (Firmicutes)**	0.001018	0.0007062	0.00009815	0.0001179	0.0139 (HF-FO vs. HF), 0.0234 (HF-EVOO vs. HF)
***Clostridium* (Firmicutes)**	0.00006808	0.0001826	0.0001873	0.0004339	0.0080 (HF-FO vs. LF)
***Coriobacteriaceae* (Actinobacteria)**	2.722e-005	0.00	0.0001803	0.0003690	0.0107 (HF-EVOO vs. HF), 0.0018 (HF-FO vs. HF)
***Anaeroplasma* (Tenericutes)**	0.005304	0.003048	0.001381	0.0002576	0.0353 (LF vs. HF-FO)

Data were analyzed using Non-parametric, Kruskal-Wallis test. Data indicate means

Supplementary Table S3 Concentrations of phenolic compounds found in EVOO and FO used in the experimental diets

Phenolic compound	EVOO Amount (ng/g)	FO Amount (ng/g)
Hydroxytyrosol	3059.29 ± 36.06	N.D.*
Tyrosol	1708.99 ± 48.55	N.D.
Vanillic acid	41.58 ± 3.70	48.98 ± 2.40
Caffeic acid	4.20 ± 0.71	1.83 ± 0.18
Homovanillic acid	N.D.	5.89 ± 0.86
p-Coumaric acid	41.59 ± 0.66	13.81 ± 0.22
Benzoic acid	N.D.	N.D.
Ferulic acid	8.88 ± 0.98	111.93 ± 5.51
o-Coumaric acid	N.D.	N.D.
Cinnamic acid	51.54 ± 3.87	6.80 ± 0.47
SDG	N.D.	N.D.
Oleuropein	N.D.	N.D.
Luteolin	542.18 ± 39.55	2.54 ± 0.20
Pinoresinol	457.151 ± 12.10	113.10 ± 2.97
Kaempferol	N.D.	1.81 ± 0.21
Apigenin	153.03 ± 4.76	2.51 ± 0.10
1-Acetoxypinoresnol	416.90 ± 16.87	N.D.
Matairesinol	N.D.	1.87 ± 0.29

*N.D. Not detected
Data were analyzed using Unpaired, Student's t test. Data indicate means ± SD. * P <0.05

Supplementary Figure S1:

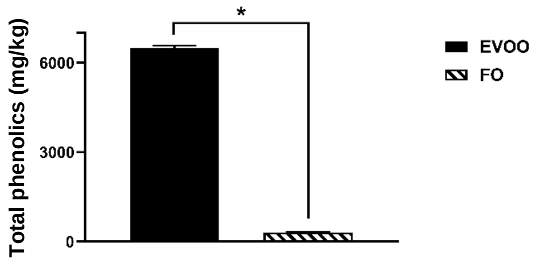

Fig. S1 Total concentration of phenolic compounds in EVOO and FO used in the experimental diets. Data were analyzed using Unpaired, Student's t test. Data indicate means ± SD. * *P* <0.05

Supplementary Figure S2:

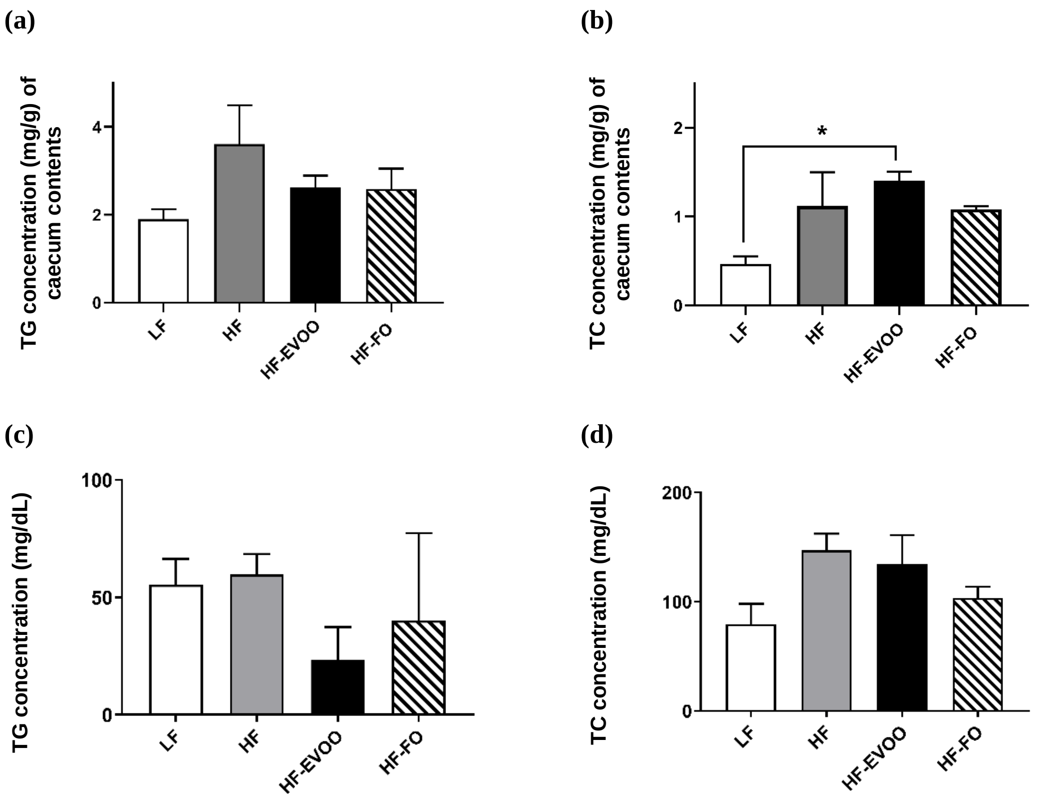

Fig. S2 (**a**) Triglyceride **(**TG) and (**b**) total cholesterol (TC) concentrations in caecum contents and (**c**) TG and (**d**) TC in plasma. Data were analyzed using one-way ANOVA followed by post hoc Tukey's multiple comparison test. Data indicate means ± SEM. n = 5 mice per group. * P <0.05

Supplementary Figure S3:

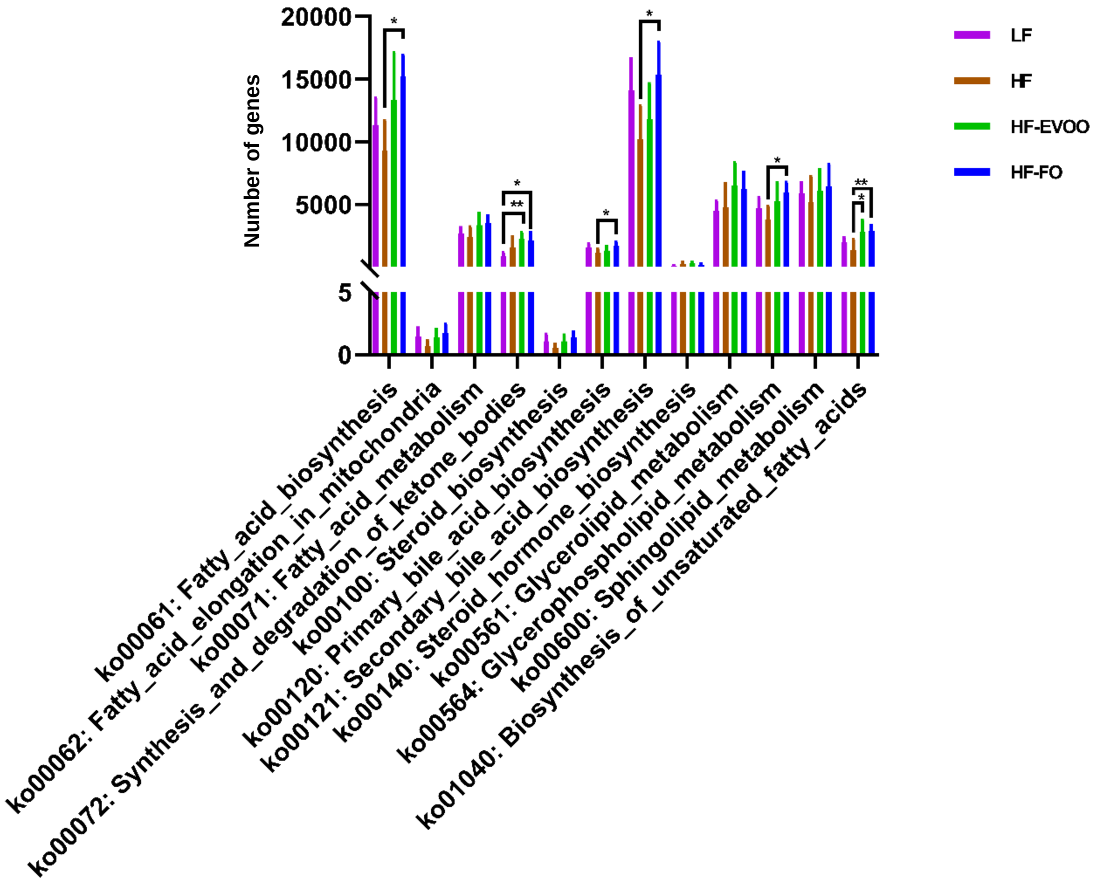

S3: Predicted metagenome functions using Kyoto Encyclopedia of Genes and Genomes (KEGG) analysis showing pathways relevant to Lipid Metabolism. Data were analyzed using one-way ANOVA followed by post hoc Tukey's multiple comparison test. Data indicate means ± SEM. $n = 5$ mice per group. * P <0.05; ** $P < 0.01$

Supplementary Figure S4:

(a)

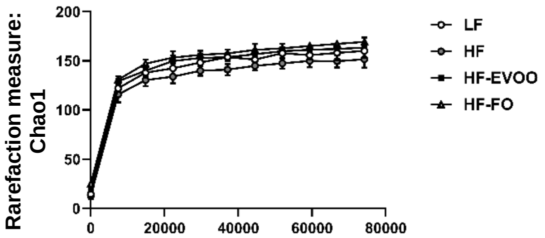

(b)

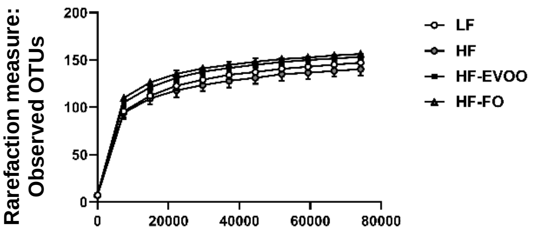

(c)

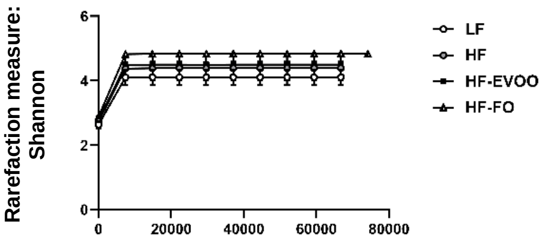

Fig. S4 Rarefaction curves displaying alpha diversity (related to Fig. 3) measured by (**a**) Chao1, (**b**) Observed OTUs and (**c**) Shannon indices in mice fed LF, HF, HF-EVOO or HF-FO. The y-axis shows diversity (units) increasing as a function of sampling depth (x-axis), with the curve plateauing when maximum sampling depth has been reached. Data indicate means ± SEM. *n* = 5 mice per group

Supplementary Figure S5:

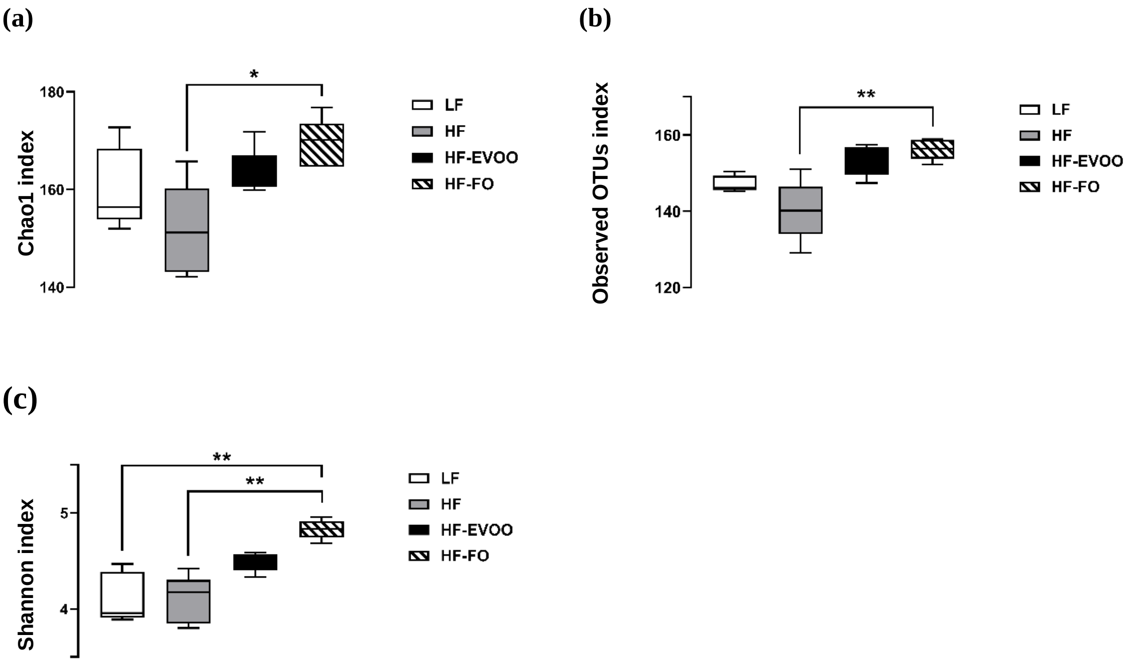

Fig S5 Box plots showing alpha diversity (related to Fig. 3) measured by (**a**) Chao1, (**b**) Observed OTUs and (**c**) Shannon indices in mice fed LF, HF, HF-EVOO or HF-FO. The y-axis shows diversity in measured units. The center line in each box plot indicates the median of the data and whiskers represent minimum and maximum values. Data were analyzed using Non-parametric, Kruskal-Wallis test. * P <0.05; ** P < 0.01

Supplementary Figure S6:

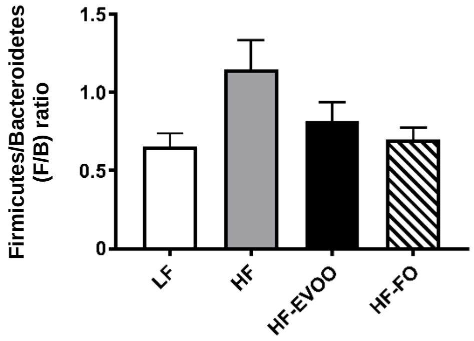

Fig S6 F/B ratio of mice fed LF, HF, HF-EVOO or HF-FO (related to Fig. 3). Data were analyzed using Non-parametric, Kruskal-Wallis test. Data indicate means ± SEM. *n* = 5 mice per group. No significant differences observed between groups

Supplementary Figure S7:

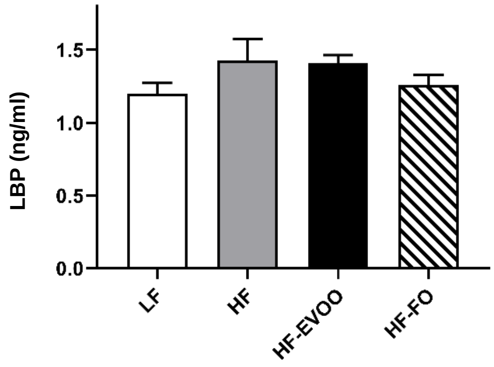

Fig S7 Concentration of lipopolysaccharide binding protein (LBP) in mouse plasma (related to Fig. 5). Data were analyzed using one-way ANOVA followed by post hoc Tukey's multiple comparison test. Data indicate means ± SEM. *n* = 5 mice per group. No significant differences observed between groups

Supplementary Figure S8:

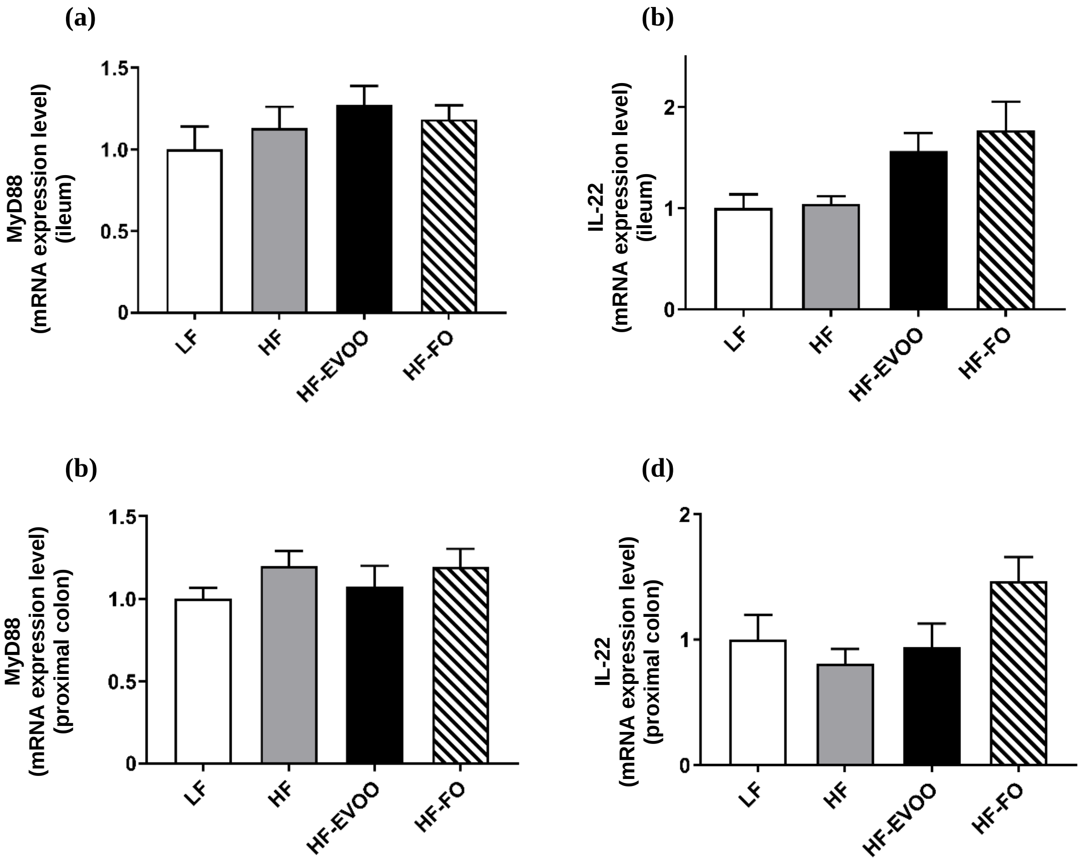

Fig. S8 Relative mRNA levels for genes involved in gut barrier defense mice fed HF, HF-EVOO or HF-FO diets as measured by q-PCR. (**a**) MyD88 and (**b**) IL-22 in ileum and (**c**) MyD88 and (**d**) IL-22 in proximal colon. Data were analyzed using one-way ANOVA followed by post hoc Tukey's multiple comparison test. Data indicate means ± SEM. *n* = 5 mice per group. No significant differences observed between groups

Supplementary Figure S9:

(a)

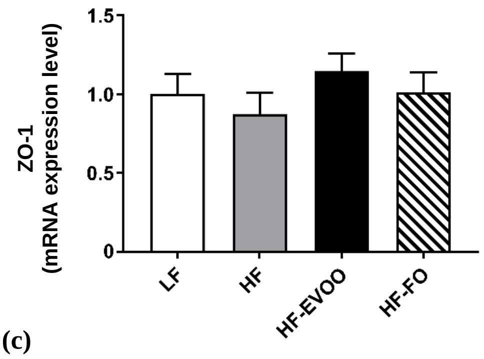

(b)

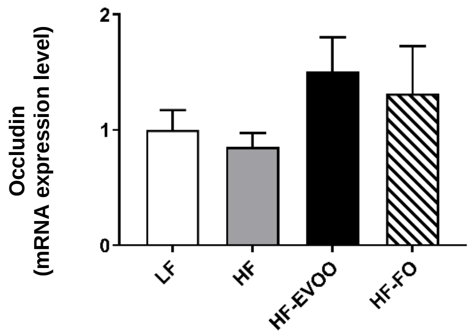

(c)

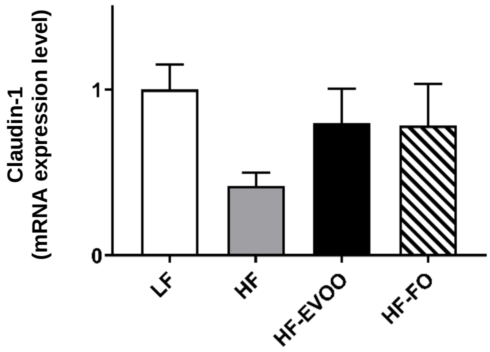

Fig. S9 Relative mRNA levels for selected gut barrier markers in proximal colon of mice fed LF HF, EVOO or FO as measured by q-PCR. (**a**) Zonula occludens-1 (ZO-1), (**b**) Occludin and (**c**) Claudin-1. Data were analyzed using one-way ANOVA followed by post hoc Tukey's multiple comparison test. Data indicate means ± SEM. *n* = 5 mice per group. No significant differences observed between groups
